# Supplementary material for: Sustained-input switches for transcription factors and microRNAs are central building blocks of eukaryotic gene circuits
Source: Genome Biol. 2013 Aug 23;14(8):R85. doi: 10.1186/gb-2013-14-8-r85 (PMC4054853; doi:10.1186/gb-2013-14-8-r85)
Supplement: Additional file 5 — HTML Browsable Motif Output. Zipped folder containing all WaRSwap and FANMOD motif output, viewable in a web browser. [file gb-2013-14-8-r85-S5.ZIP › HTML_browsable_motif_output/WaRSwap_ath_tair9/sigs_WSWRSLDraw_2-2000.pvals.heatmaps.html/motif_id_46_011101001_tftype_ath_upstream_-1000_0.html]

```
BG_MODEL = Rnew2
MOTIF_ID = 46_011101001
TF_TYPE = ath
UPSTREAM = -1000_0


PVals
FN_0.2	FN_0.4	FN_0.6	FN_0.8
dg_60.genes	0.07	0.006	0.243	0.267
dg_70.genes	0.069	0.009	0.243	0.264
dg_80.genes	0.066	0.007	0.253	0.275

ZScores
FN_0.2	FN_0.4	FN_0.6	FN_0.8
dg_60.genes	1.398	2.439	0.534	-0.46
dg_70.genes	1.38	2.493	0.544	-0.45
dg_80.genes	1.388	2.509	0.526	-0.45

StDevs
FN_0.2	FN_0.4	FN_0.6	FN_0.8
dg_60.genes	893.31	318.952	29.829	2.944
dg_70.genes	866.174	313.594	30.16	2.897
dg_80.genes	875.609	310.248	30.14	2.829
```
